# Trends in Medical School Applications and Acceptances From Historically Black Colleges and Universities, 1980-2022

**DOI:** 10.1001/jamanetworkopen.2025.22154

**Published:** 2025-07-21

**Authors:** Jasmine Weiss, Evan Galloway, Sai Meghana Mugi, Jessica Young, Erin Fraher, Darin Latimore, Inginia Genao

**Affiliations:** 1 Division of General Pediatrics and Adolescent Medicine, Department of Pediatrics, University of North Carolina School of Medicine, Chapel Hill; 2 Cecil G. Sheps Center for Health Services Research, University of North Carolina at Chapel Hill, Chapel Hill; 3 University of North Carolina Gillings School of Public Health, Chapel Hill; 4 Departments of Family Medicine, University of North Carolina at Chapel Hill School of Medicine; 5 Department of Internal Medicine, Yale University School of Medicine, New Haven, Connecticut; 6 Department of Medicine, Penn State College of Medicine, Hershey, Pennsylvania

## Abstract

**Importance:**

Historically Black Colleges and Universities (HBCUs) play a pivotal role in higher education and the physician career path in the US. Studies have highlighted the importance of HBCU medical schools in the training of Black physicians. However, little is known about the contributions of HBCU undergraduate institutions to the medical school pathway over the past 30 years.

**Objective:**

To describe trends in Black HBCU undergraduate students’ medical school application and acceptance rates from 1980 to 2022.

**Design, Setting, and Participants:**

This descriptive cross-sectional study analyzed data from the Association of American Medical Colleges from 1980 to 2022 on medical school applicantions and acceptances, as well as National Center for Education Statistics data on Black bachelor’s degree recipients (1990-2020). Data were analyzed from August to December 2023.

**Main Outcomes and Measures:**

The number and proportion of medical school applicants and accepted students were compared over time for Black students who completed their undergraduate training at HBCUs vs non-HBCUs.

**Results:**

The study population included 1 666 145 total applicants (754 580 female [45.3%]; 146 852 Black [8.8%]). Between 1990 and 2020, Black bachelor’s degree recipients from HBCUs increased by 50% (from 17 930 to 26 944 recipients), while those from non-HBCUs rose 271% (from 48 445 to 179 583 recipients). Black representation among medical school applicants grew from 7.2% in 1980 (2490 of 34 369 applicants) to 11.5% (5895 of 51 385 applicants) in 2022. From 1990 to 2017, Black HBCU graduates made up a higher proportion of Black medical school applicants, but from 2018 to 2020, non-HBCU graduates contributed more to the applicant pool. Throughout the study period, Black HBCU applicants had consistently lower medical school acceptance rates than their non-HBCU counterparts.

**Conclusions and Relevance:**

This cross-sectional study found that while HBCUs consistently produced a significant portion of Black medical school applicants, Black HBCU graduates faced lower medical school acceptance rates compared with their non-HBCU peers. Underinvestment in HBCUs and potential biases within medical school admissions may contribute to these challenges, limiting the full potential of HBCU students in diversifying the physician workforce; addressing these disparities through HBCU support, partnerships, and funding could enhance pathways to medical careers for Black students and contribute to achieving greater health equity.

## Key Points

**Question:**

What are the trends in medical school application and acceptance rates for Black undergraduate students who graduated from Historically Black Colleges and Universities (HBCUs)?

**Findings:**

This cross-sectional study of 1 666 145 medical school applicants found that the number of Black medical school applicants increased longitudinally from 1980 to 2022. Black HBCU graduates represented a higher proportion of Black applicants from 1990 to 2017 compared with 2018 to 2020 but consistently had lower medical school acceptance rates than non-HBCU Black graduates.

**Meaning:**

Black HBCU students contribute substantially to the physician training pathway, and further investigation is needed to explore why Black HBCU students apply at a higher rate but are accepted to medical school less often.

## Teaser

This cross-sectional study analyzes rates of medical school application and acceptance among Black undergraduates from Historically Black Colleges and Universities (HBCUs) from 1980 to 2022.

## Introduction

Black Americans continue to face disparities in health care access, self-reported health status, and many other health outcomes.^1,2,3^ Black patients with Black physicians are more likely to receive preventive services and adhere to medication regimens.^4,5^ Studies highlight an association of lower infant mortality rates among Black newborns with being cared for by Black physicians.^6^ Additionally, higher concentrations of Black primary care physicians in a county is associated with increased life expectancy and lower all-cause mortality.^7^ Patient satisfaction and communication improves when patients and clinicians share similar racial and ethnic backgrounds.^8,9^ Furthermore, Black physicians are more likely to accept uninsured and Medicaid patients and express a desire to practice in underserved communities.^10^

Despite these positive contributions to improving health outcomes, Black physicians remain underrepresented in medicine (URiM). Individuals are considered URiM if they belong to racial and ethnic groups with less representation in the medical profession relative to the general population. Black Americans account for 13.6% of the US population; however, only 5.7% of all physicians are Black.^11^ Despite efforts such as Project 3000 by 2000 aimed at increasing the representation of URiM students admitted to medical school and ultimately growing the physician workforce, progress has been marginal over the past decades.^12,13^

Historically Black Colleges and Universities (HBCUs) have been a cornerstone of higher education for Black Americans since their inception postslavery.^14^ HBCUs only make up 3% of all 4-year degree granting colleges, yet they educate 10% of Black students pursuing bachelor’s degrees, produce 20% of Black graduates completing bachelor’s degrees, and 25% of Black undergraduate students pursuing science, technology, engineering, or mathematics degrees.^15,16^ HBCU undergraduate institutions have substantially contributed to the physician medical education pathway, but their impact has been understudied.^17^ This descriptive study narrows this gap by exploring trends in medical school application and acceptance rates of students who self-identify as Black and attended HBCUs for undergraduate training.

Striving to achieve health equity by increasing representation throughout the medical education pathway requires increasing the cultural sensitivity of the workforce to provide care to diverse patient populations.^18,19,20^ Understanding the trends in medical school admissions for Black HBCU students can help identify potential opportunities to increase Black students entering the physician medical education pathway and, ultimately, the physician workforce. In the context of the 2023 Supreme Court race-neutral admissions ruling (Students for Fair Admissions vs Harvard and Students for Fair Admissions vs University of North Carolina)^21^ and the decline that some predominantly White institutions (PWIs) are already seeing in diversity in their incoming classes,^22^ it is imperative to understand the impact of HBCUs in contributing to the medical school applicant pool for Black students. This study examined the following question: What are the trends in medical school application and acceptance rates of students who self-identify as Black and attended HBCUs for undergraduate training?

## Methods

This cross-sectional study utilized AAMC data (1980 to 2022) from the American Medical College Application Service (AMCAS), including student demographic characteristics, and application year. Data from the AAMC Student Records System included medical school enrollment year and medical school graduation status (reported as graduating, yes or no). Inclusion criteria were US citizens who self-identified as Black or African American alone or in combination with other racial and ethnic groups and those who applied to a 4-year degree-granting allopathic medical school. This study was deemed exempt by the University of North Carolina at Chapel Hill Office of Human Research Ethics because the study involved nonhuman participant research and data were deidentified. The study followed the Strengthening the Reporting of Observational Studies in Epidemiology (STROBE) reporting guideline.^23^

We used race and ethnicity categorizations defined by the AAMC. Throughout the study period, the AAMC made 2 changes to race and ethnicity data collection. From the 1980 to 1981 academic year until the 2001 to 2002 academic year, individuals could only select 1 race or ethnicity category. From the 2002 to 2003 academic year through the 2012 to 2013 academic year, the AAMC utilized 2 separate questions; the first asked about race and the second asked about Hispanic origin. Respondents could select more than 1 response option. From the 2013 to 2014 academic year until the present, the AAMC uses 1 question to collect race and ethnicity data. The race and ethnicity categories that individuals could select included: American Indian or Alaska Native; Asian; Black or African American; Hispanic, Latino, or of Spanish Origin; Native Hawaiian or Other Pacific Islander; White; and other race or ethnicity. This single question allowed individuals to select from multiple race and Hispanic ethnicity categories.^24^ We categorized respondents as Black if they self-identified as Black or African American alone or in combination with other racial and ethnic groups, given the changes in the AAMC race and ethnicity data collection structure during the study period. We excluded individuals with missing race and ethnicity or sex data, non-US citizens, and medical school matriculants who transferred from other health professional schools because they did not submit their applications through AMCAS and were therefore missing key application and acceptance data.

This study explored trends in medical school application and acceptance rates of self-identified Black students who attended HBCUs for their undergraduate training compared with those who attended non-HBCUs. Outcomes of interest included the number and proportion of Black students who applied to medical school and were accepted each year from 1980 to 2022. We categorized students by their AMCAS application year and medical school graduation status (yes or no). Graduation data were only available from 1980 to 2017. Key variables included the undergraduate institutional designation of HBCU vs non-HBCU and race and ethnicity (Black alone or in combination). Applicants missing the HBCU designation variable were assigned the non-HBCU category. Non-HBCU institutions include PWIs, Hispanic-serving institutions, and Native-serving institutions. We obtained data on the number of Black bachelor’s degree recipients from 1990 to 2020 from the US Department of Education, Institution of Education Sciences, collected as part of the Integrated Postsecondary Education Data System, a mandatory annual survey of all postsecondary educational institutions in the US that participate in federal financial aid programs.

### Statistical Analysis

This study analyzed a subset of students who self-identified as Black or African American (alone or in combination with other race and ethnicity groups). We compared (1) the percentage of Black bachelor’s degree recipients who applied to medical school from HBCUs vs non-HBCUs over time and (2) the percentage of Black medical school applicants accepted to medical school from HBCUs vs non-HBCUs over time. Statistical analysis was conducted from August to December 2023 using Stata 17 (StataCorp).

## Results

The study population included 1 666 145 total applicants (754 580 female [45.3%]; 146 852 Black alone or in combination [8.8%]). There were 146 852 applicants who self-identified as Black alone or in combination with other racial and ethnic groups.

### Black Bachelor’s Degree Recipients

**Figure 1.**
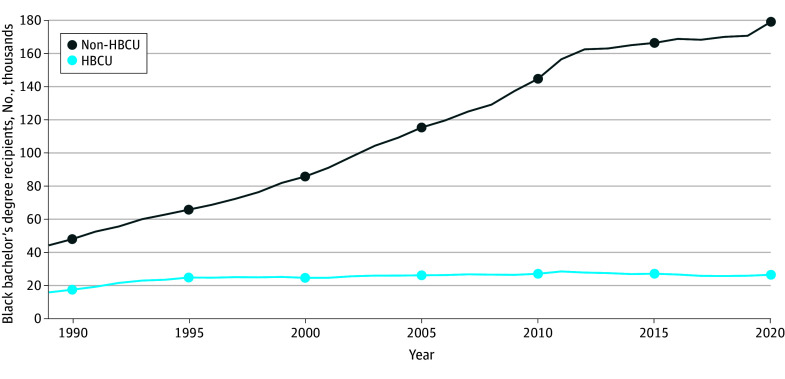
Black Bachelor’s Degree Recipients by Institutional Designation (1989-2020) Data Source: US Department of Education: Institute of Education Sciences.^25^ HBCU indicates Historically Black Colleges and Universities.

Black students earning bachelor’s degrees steadily increased during the study period, rising from 66 375 graduates to 206 527 graduates (Figure 1). The number of Black students receiving bachelor’s degrees from non-HBCUs rose over the past 30 years. In the 1990 to 1991 academic year, Black students received 48 445 of 66 375 bachelor’s degrees (73.0%) from non-HBCUs, increasing to 179 583 of 206 527 bachelor’s degrees (87.0%) in 2020 (a 271% increase).

While the number of students earning bachelor’s degrees from HBCUs increased from 17 930 recipients in 1990 to 26 944 recipients in 2020 (a 50% increase), the proportion of all Black graduates declined from 27.0% (17 930 of 66 375 graduates) to 13.0% (26 944 of 206 527 graduates). At its peak, 15.6% of Black bachelor’s degree recipients (28 944 of 185 916 recipients) earned their degrees from HBCUs in the 2011 to 2012 academic year.

### Black Medical School Applicants

**Figure 2.**
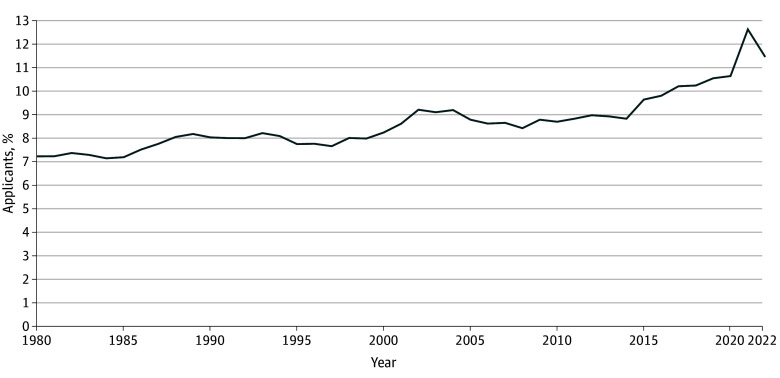
Percentage of Black Medical School Applicants (1980-2022) Data Source: Association of American Medical Colleges, collected as part of the Integrated Postsecondary Education Data System.^25^

The increasing trend in Black students receiving bachelor’s degrees coincided with a rising number of Black medical school applicants. Black students represented 7.2% of all medical school applicants (2490 of 34 369 applicants) in 1980 and trended upward to 11.5% (5895 of 51 385 applicants) in 2022 (Figure 2). Notably, in 2021, Black students represented 12.6% of all medical school applicants (7279 of 57 572 applicants)—the highest number of Black applicants observed during the study period. This was followed by a subsequent decline for Black medical school applicants to 11.5% (5895 of 51 385 applicants) in 2022. In 2021, the total number of applications from all applicants increased by 18.9%, from 48 433 applications in 2020 to 57 572 applications in 2021. This was accompanied by a 10.7% decline of total applicants to 51 385 applicants in 2022. This spike coincided with the COVID-19 pandemic and a broader increase in applicants across all racial and ethnic groups. Expanded fee assistance programs for the Medical College Admission Test (MCAT) and medical school applications, along with other temporary changes to the admissions process in the setting of the COVID-19 pandemic, may have also contributed to this increase.^26^

**Figure 3.**
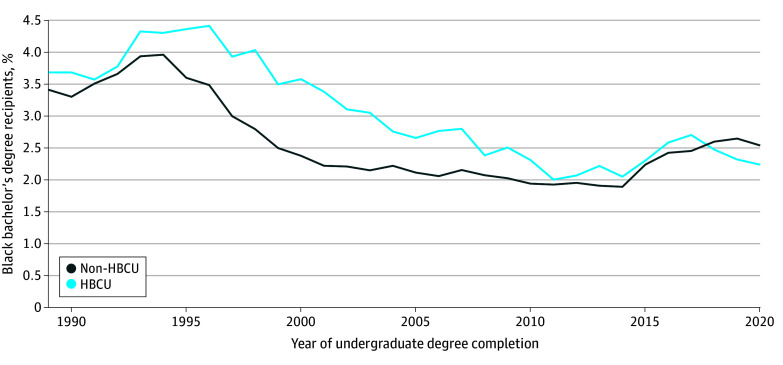
Percentage of Black Bachelor’s Degree Recipients Who Applied to Medical School by Institutional Designation (1989-2020) Data Source: Association of American Medical Colleges and National Center for Education Statistics, collected as part of the Integrated Postsecondary Education Data System.^25^

The proportion of Black students applying to medical school from HBCUs vs non-HBCUs changed over time. Between 1990 to 2017, a larger percentage of Black HBCU graduates applied to medical school than Black graduates of non-HBCUs. This reached its peak in 1996 when 1110 of 25 168 Black HBCU graduates (4.4%) applied to medical school (Figure 3). Beginning in 2018, Black bachelor’s degree recipients from non-HBCU institutions represented a greater proportion of Black medical school applicants.

### Medical School Acceptance Rates for Black Students

**Figure 4.**
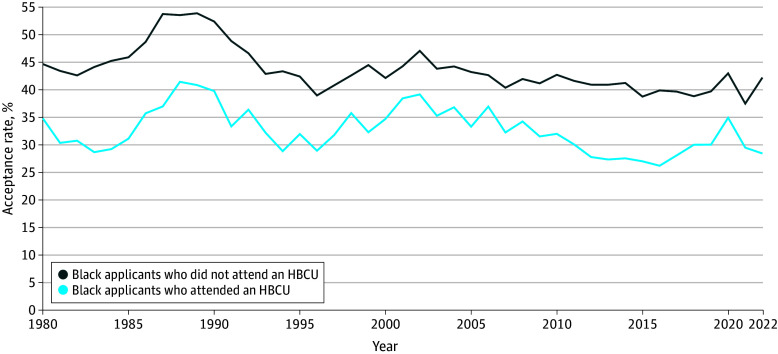
Medical School Acceptance Rate by Institutional Designation, 1980-2022 Data Source: Association of American Medical Colleges, collected as part of the Integrated Postsecondary Education Data System.^25^ Black applicants included those who identified as Black or African American alone or in combination with other racial and ethnic groups. HBCU indicates Historically Black Colleges and Universities.

The acceptance rate for Black HBCU students remained consistently lower than for Black non-HBCU students throughout the study period (Figure 4). However, the acceptance rate of Black students from HBCUs and non-HBCUs fluctuated from 1980 to 2022. It peaked in 1988 when 235 of 566 Black students (41.5%) accepted to medical school were from HBCUs. For Black non-HBCU students, the highest acceptance rate occurred in 1987 when 822 of the 1527 applicants (53.8%) were accepted. The lowest acceptance rate for Black HBCU applicants occurred in 2016, with only 184 of 700 applicants (26.3%) being accepted. In contrast, the year with the lowest acceptance rate for Black non-HBCU applicants occurred in 2015, when 1451 of 3736 applicants (38.8%) were accepted into medical school.

## Discussion

Ensuring that the physician workforce reflects the diversity of the US population is critical for addressing and alleviating health disparities in marginalized communities. HBCUs have played a crucial role in educating Black students in preparation for physician careers.^17,27,28^ This cross-sectional study examined trends in medical school application and acceptance of Black students from HBCUs and non-HBCUs. While HBCUs continue to produce many Black medical school applicants, our findings highlight the disparity in medical school acceptance rates. Throughout the study period, Black HBCU undergraduate students consistently had lower medical school acceptance rates than their non-HBCU peers.

**Figure 5.**
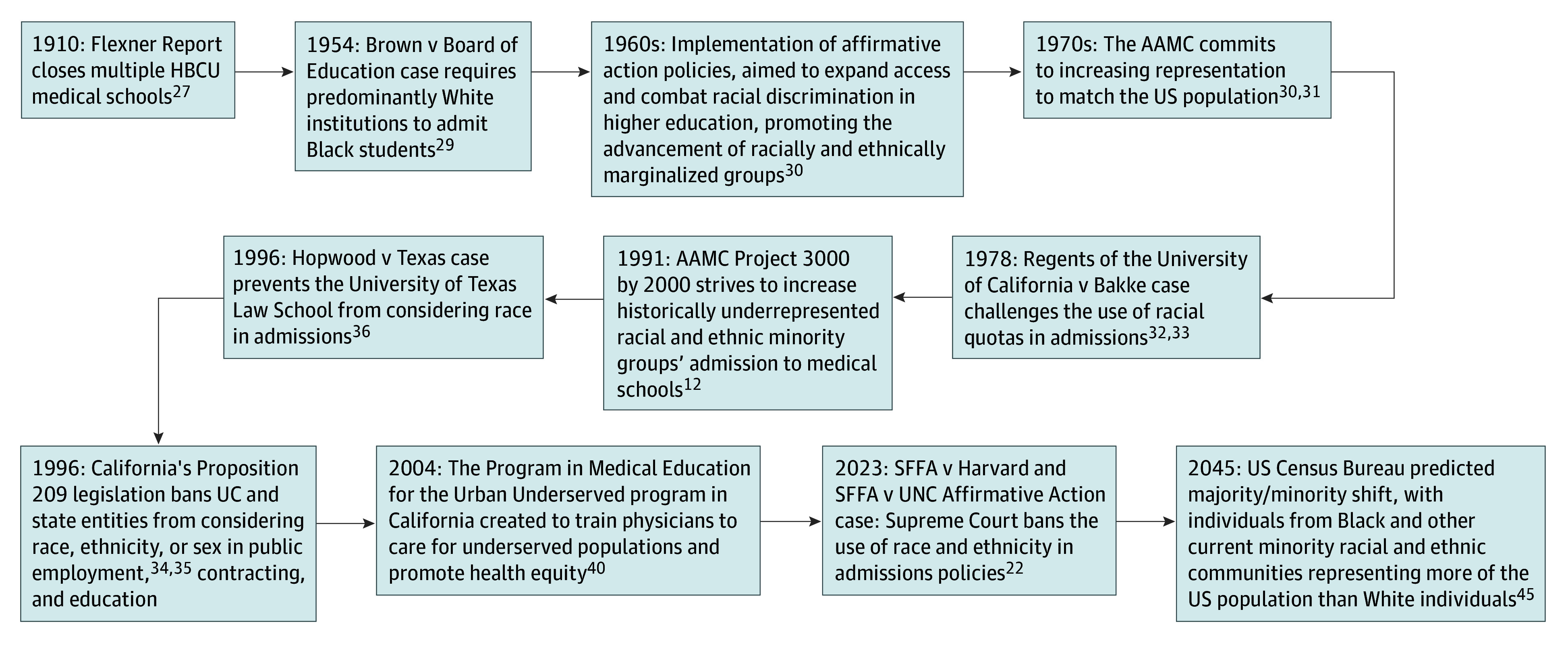
Historical Milestones Shaping Medical Student Diversity AAMC indicates Association of American Medical Colleges; HBCU, Historically Black Colleges and Universities; SFFA, Students for Fair Admissions; UC, University of California; UNC, University of North Carolina.

These findings raise concern about the complex interplay between systemic, institutional, and individual racism that contributes to the persistent underrepresentation of Black physicians. Thirteen HBCU medical schools existed in the late 1800s and early 1900s.^29^ The Flexner Report of 1910 aimed to standardize medical education, but simultaneously limited opportunities for Black students^30^ (Figure 5). Flexner recommended that Black individuals refrain from training as surgeons and specialists but instead focus on hygiene and the prevention of disease spread.^31^ Flexner also recommended that 5 of the 7 HBCU medical schools that were operational at the time close. These HBCU medical school closures had lasting consequences on the production of Black physicians. Studies estimate a projected loss of an additional 35 000 Black physicians graduating from HBCU medical schools due to their closure following the Flexner Report.^29^ More HBCU medical schools would increase opportunities for medical school admission overall, but particularly for affiliated HBCU undergraduate institutions, given the proximity on the same campuses and access to physicians for mentorship, shadowing, and research opportunities. Our findings suggest that these historical decisions, which affected all Black students, may have had a more pronounced impact on Black students attending HBCUs.

Before the landmark case of Brown vs Board of Education (1954), which required PWIs to admit Black students, HBCUs enrolled more than 90% of all Black students.^32^ The implementation of affirmative action policies in the 1960s and the AAMC commitment to increasing representation to match the US population in the 1970s led to initial increases overall in enrollment for Black individuals in higher education.^33,34^ However, this consequently led to fewer students attending HBCUs because they gained access to other institutions. These efforts ultimately did not achieve population parity in the physician workforce in the wake of Supreme Court decisions like Regents of the University of California vs Bakke (1978), which challenged the use of racial quotas in admissions.^35,36^

This trend in medical education governing bodies advocating for increased representation followed by legislative policies to substantially hinder such efforts has continued to occur in an oscillatory fashion. The 1990s AAMC Project 3000 by 2000^12^ to increase underrepresented racial and ethnic minorities’ admission to medical school was adversely impacted by California Proposition 209 legislation in 1996,^37,38^ Hopwood vs Texas (1996),^39^ and other ballot initiatives restricting race-conscious admissions.^40^ Initiatives such as holistic review, aimed at assessing the whole applicant including their unique background, attributes, and experiences along with their academic metrics were created to take into account more than just meritocratic grades and test scores and to view life experiences and background more equally when assessing the fit of applicants for consideration for medical school admission.^13,41,42^ The Program in Medical Education for the Urban Underserved program in California was created to “recruit and train physicians to care for underserved populations, expand the healthcare workforce to serve diverse populations, and promote health equity.”^43^ Despite these initiatives to recruit individuals to the physician workforce and more broadly to promote health equity in underserved communities, recent legislation, such as the Supreme Court decision in 2023 which bans the use of race and ethnicity in admissions policies, brings medical education back to a crossroads that may have consequences for URiM students for years to come.^21^ Race-neutral admissions have already shown early consequences for Black individuals pursuing medical school with a 2.8% increase of Black applicants 2024 but an 11.6% decline in Black matriculants in the same cycle, according to the AAMC.^44^ This finding parallels declines observed in California following Proposition 209 with the proportion of racially and ethnically minoritized matriculants dropping from 23.1% to 14.3% (a decline of 8.7%) between 1993 and 1997.^45^ These cyclical declines following the passage of restrictive legislation highlight the critical role of HBCUs in supplying qualified medical school applicants and supporting students along the pre-medical pathway.

Despite the overrepresentation of Black HBCU graduates in the medical school applicant pool for a substantial portion of our study period, our findings demonstrate a longstanding discrepancy in medical school acceptance rates for Black HBCU graduates. This multifactorial disparity may be attributed to several intersecting factors including differences in academic metrics, limited institutional supports at some HBCUs, and implicit or explicit bias within the medical school admissions process. Some studies highlight that MCAT scores and grade point average (GPA) are overemphasized in medical school admissions, which disproportionately disadvantages Black applicants.^41,46^ Our findings highlight that this may more drastically impact Black HBCU students who may face disadvantages in accessing resources necessary to achieve what are often considered competitive MCAT scores and GPAs.

Another factor that may contribute to the disparity in acceptances for Black HBCU students compared with non-HBCU students includes a lack of institutional support or infrastructure to provide opportunities for students at some HBCUs.^47^ The Department of Education reports funding disparities of more than 1 billion dollars for multiple land-grant HBCUs across decades.^48^ Even at the same research ranking, HBCUs continue to receive less funding than PWIs.^48,49^ These funding gaps may contribute to the lack of infrastructure at some HBCUs limiting students’ access to research and clinical shadowing opportunities, MCAT preparation resources, and dedicated advising.^47^ These disparities in institutional funding highlight the need for increased federal, state, philanthropic, and alumni financial resources to ensure that HBCU students can optimally prepare for the medical school admissions process and ultimately contribute to the physician workforce.

Our study raises concerns for the role of medical schools and their disparate selection of Black students from HBCUs compared with students from non-HBCUs. These findings could be explained by implicit or explicit biases against HBCUs and may contribute to lower medical school acceptance rates for HBCU students. Studies on racial salience suggest that individuals may hold biased viewpoints toward Black students from HBCUs, perceiving them as having a stronger racial identity compared with those from non-HBCU backgrounds.^50,51^ PWI medical school admissions committees should consider if lack of familiarity with HBCUs, their history, and culture, may inadvertently influence admissions decision-making beyond academic metrics alone.^52,53^ Because relatively fewer admissions committee members have personally attended HBCUs, they might have biases about the quality or rigor of the education provided and thus the preparedness of their graduates.

This declining trend in Black HBCU graduates being accepted despite applying to medical school highlights the need for medical schools to closely evaluate their commitment to creating the most diverse medical school classes possible to keep pace with the growing racially and ethnically minoritized population in the US. By the year 2045, there will be a majority-minority shift, according to the US Census Bureau, with individuals from racially or ethnically marginalized communities representing more of the US population than White individuals.^54^ To reverse this trend, medical schools should consider partnerships with HBCUs for research, shadowing, and mentorship programs for premed students. Articulation agreements and early enrollment programs should be considered to create more opportunities for HBCU students who may have limited access to resources at their home institutions.^47^ Considerations for postbaccalaureate programs at HBCUs may also be useful for assisting URiM students with gaining admissions into medical school because these programs expose students to the academic rigors of a medical school curriculum while helping to refine academic and study skills. Medical schools should also critically examine their admissions policies to identify potential biases against Black students attending HBCUs for their undergraduate degrees. With physician shortages looming by 2036,^55^ medical schools must critically examine their use of holistic review when considering medical school admissions. HBCUs have the potential to contribute substantially to ensuring that Black individuals continue to bolster the country’s medical student body and physician workforce.

Our findings underscore the need for a collective effort to support and bolster the students from HBCUs seeking medical school admission and, ultimately, careers as physicians. HBCUs should advocate for additional state funding to support expanded offerings for MCAT test preparation, premedical advising, research, and mentorship. The federal government and states should award more funding to support HBCU institutions, especially in creating and supporting future physicians amid a looming shortage of physicians. Additionally, support should be given for undergraduate HBCUs such as Xavier University and Morgan State University, which are currently establishing new Black medical schools.^56,57^ Consideration should be given for funding to expand the class sizes at the current Black medical schools, (Morehouse School of Medicine, Meharry Medical College, Howard University College of Medicine, and Charles R. Drew University of Medicine and Science), which have collectively trained more than 50% of Black medical school graduates.^58^

To further support HBCU students, national organizations such as the AAMC, American Association of Colleges of Osteopathic Medicine, and the Liaison Committee on Medical Education should consider developing targeted programs to assist HBCU students in navigating the medical school application process. This comprehensive approach, engaging national organizations, medical schools, and HBCUs, is necessary to create a more equitable pathway for Black HBCU students to have physician careers (eTable in Supplement 1).

### Limitations

This study has limitations. The sample excluded physicians applying to osteopathic programs. Future studies will be needed to address differences in medical school attrition and medical school graduation for Black students who completed their undergraduate training at HBCUs compared with those who completed their undergraduate training at non-HBCUs. Future studies will be needed to explore other factors that may contribute to these trends, such as taking gap years before applying to medical school, financial barriers, individual institutional characteristics, student academic performance (GPA and MCAT scores), and individual socioeconomic status (ie, parental income).

## Conclusions

This study highlights the role of HBCUs in supplying students to the medical school pipeline and ultimately contributing to a more diverse medical student body and physician workforce in the US. Our study identified significant disparities in medical school application and acceptance rates among Black students from HBCUs compared with Black students from non-HBCU institutions. HBCU graduates face consistently lower medical school acceptance rates despite representing a substantial portion of Black applicants. These findings underscore Black HBCU students’ systemic challenges in accessing medical education. As a result, it emphasizes the importance of addressing both historical underinvestment^41,42^ in HBCUs and potential biases within medical school admissions.^43^ Increasing the representation of Black physicians in the workforce is critical to achieving health equity for historically marginalized communities. Future research should explore factors contributing to the acceptance disparities for HBCU graduates. Additionally, studies should examine the impact of targeted interventions in supporting these students, such as partnerships and mentoring programs between HBCUs and medical schools. A collaborative approach involving institutions, policymakers, and accrediting bodies is necessary to create a more inclusive pathway into medicine. Ultimately, this will enhance the health care system’s capacity to meet the needs of an increasingly diverse population.

## Notes

Supplement 1. **eTable.** Recommendations to Strengthen the Medical School Pathway for HBCU Undergraduate Students

Supplement 2.
Data Sharing Statement
