# Supplementary material for: Trends in Medical School Applications and Acceptances From Historically Black Colleges and Universities, 1980-2022
Source: JAMA Netw Open. 2025 Jul 21;8(7):e2522154. doi: 10.1001/jamanetworkopen.2025.22154 (PMC12281238; doi:10.1001/jamanetworkopen.2025.22154)
Supplement: Supplement 1. — eTable. Recommendations to Strengthen the Medical School Pathway for HBCU Undergraduate Students [file jamanetwopen-e2522154-s001.pdf]

Supplemental Online Content

Weiss J, Galloway E, Mugi SM, et al. Trends in medical school applications and acceptances from Historically Black Colleges and Universities, 1980-2022. *JAMA Netw. Open*. *JAMA Netw. Open*. 2025;8(7):e2522154. doi:10.1001/jamanetworkopen.2025.22154

eTable. Recommendations to Strengthen the Medical School Pathway for HBCU Undergraduate Students

This supplemental material has been provided by the authors to give readers additional information about their work.

© 2025 Weiss J et al. *JAMA Network Open*.

eTable: Recommendations to Strengthen the Medical School Pathway for HBCU Undergraduate Students

Adapted from: Weiss J, Nguementi Tiako MJ, Akingbesote ND, et al. Perspectives on medical school admission for Black students among premedical advisers at historically Black colleges and universities. JAMA Netw Open. 2024;7(10):e2440887. doi:10.1001/jamanetworkopen.2024.40887

Stakeholder Group	Recommendation	Description
HBCU Undergraduate Institutions	Increase MCAT preparation support*	Provide accessible MCAT prep through workshops, tutoring, and subsidized test prep materials.
	Expand clinical exposure pathways	Support diverse forms of clinical experience, including allied health certifications, medical scribing, and interdisciplinary internships.
	Expand tutoring infrastructure	Offer additional subject-specific tutoring for core premedical courses (eg, organic chemistry, biology) to strengthen academic readiness.
	Advocate for state and federal funding*	Advocate for increased funding to support premedical offices, scholarships, and research infrastructure.
	Promote early exposure opportunities	Develop pipeline programs from high school to college in collaboration with community organizations.
	Leverage peer and near-peer support	Create structured mentorship networks among students and alumni to foster accountability, guidance, and community.
Medical Schools	Strengthen institutional partnerships	Establish equitable collaborations with HBCUs to offer mentorship, research access, and interview preparation.
	Broaden evaluation metrics*	Apply holistic review by valuing life experiences, perseverance, and community engagement alongside academic performance.
	Offer transparent recruitment and selection*	Publish data on HBCU applicant outcomes and formalize recruitment through articulation agreements.

© 2025 Weiss J et al. *JAMA Network Open*.

	Provide summer programs and bridge opportunities	Develop targeted summer enrichment or postbaccalaureate programs to strengthen HBCU student competitiveness.
HBCU Medical Schools	Advocate for funding to Increase medical school enrollment*	Advocate for increased state and federal funding to increase enrollment size fo incoming classes
Funders, Policymakers, and National Organizations	Develop a national shadowing platform	Support a centralized digital tool to connect URiM students with physicians for shadowing experiences nationwide.
	Support virtual clinical exposure*	Endorse virtual shadowing and telehealth-based experiences to overcome geographic access barriers.
	Fund HBCU capacity building*	Allocate resources toward MCAT prep, research infrastructure, and faculty development at HBCUs.
	Consider data transparency in admissions*	Require disaggregated reporting of URiM and HBCU applicant outcomes across all medical schools.

*Recommendations proposed by the study team. All others were derived from adviser perspectives reported in Weiss J, et al. Perspectives on Medical School Admission for Black Students Among Premedical Advisers at HBCUs. JAMA Netw Open. 2024.

© 2025 Weiss J et al. *JAMA Network Open*.
