# Supplementary material for: Trends in Medical School Applications and Acceptances From Historically Black Colleges and Universities, 1980-2022
Source: JAMA Netw Open. 2025 Jul 21;8(7):e2522154. doi: 10.1001/jamanetworkopen.2025.22154 (PMC12281238; doi:10.1001/jamanetworkopen.2025.22154)
Supplement: Supplement 2. — Data Sharing Statement [file jamanetwopen-e2522154-s002.pdf]

Data Sharing Statement

Weiss. The Contributions of Historically Black Colleges and Universities on Diversifying the Medical Student Body. *JAMA Netw Open*. Published July 21, 2025. doi:10.1001/jamanetworkopen.2025.22154

Data
Data available: No
